# Indicators of adherence to physiotherapy attendance among Saudi female patients with mechanical low back pain: a clinical audit

**DOI:** 10.1186/1471-2474-11-124

**Published:** 2010-06-17

**Authors:** Einas Al-Eisa

**Affiliations:** 1 Department of Health Rehabilitation, College of Applied Medical Sciences, King Saud University, Riyadh, Saudi Arabia

## Abstract

**Background:**

Among current musculoskeletal interventions used to treat low back pain (LBP), physiotherapy exercise has the highest evidence of effectiveness in avoiding recurrence and chronic disability. However, effectiveness of physiotherapy is thought to be directly related to the patients' adherence to physiotherapy. Since adherence is reported to be directly influenced by socio-cultural factors, this study was conducted to investigate factors related to patients' adherence in a group of Saudi female patients with LBP.

**Methods:**

A retrospective chart review was conducted on female LBP patients referred to the department of physiotherapy at a local tertiary hospital over a 12 month period. A total of 98 charts were reviewed. Two physiotherapists specialized in musculoskeletal rehabilitation collected information from the medical files. Data were classified in three categories: patients' personal demographics, patients' medical condition and history, and type of physiotherapy administered. Contingency tables and chi-square test were computed to test for differences in proportions. Spearman rank correlation coefficient was calculated to examine relationships among variables.

**Results:**

Subjects who attended their scheduled appointments were classified as adherent (40%), and those who failed to attend 2 consecutive scheduled appointments and got discharged were classified as non-adherent (60%). Factors that significantly correlated with adherence included: age (r = 0.7, p < 0.05), initial pain intensity (r = 0.5, p < 0.05), and subjective report of improvement (r = 0.7, p < 0.01). Adherence did not correlate with the type of LBP, patient occupation, experience or nationality of the physiotherapist.

**Conclusion:**

This study reveals an alarming level of non-adherence to physiotherapy among patients with LBP. It remains unclear as to what level of adherence is required to achieve beneficial effect of treatment. It is quite evident however, that early withdrawal from treatment would not allow the therapeutic benefits of the treatment to be realized. Future research should be directed toward developing strategies to improve adherence.

## Background

Low back pain (LBP) is considered a major health problem due to its high prevalence [1,2], high probability of recurrence [3], and associated disability [4]. It is generally defined as the perception of pain in the posterior aspect of the body between the inferior border of the rib cage and the inferior gluteal fold [5]. The epidemiology and socioeconomic cost of LBP has been well documented [6-8]. The majority of back pain sufferers seek conservative treatment such as physiotherapy [9]. In fact, LBP may account for over 50% of referrals to out-patient physiotherapy departments [10].

There is a growing amount of literature examining the effectiveness of existing conservative approaches to management of back pain [11,12]. Among current musculoskeletal interventions used to treat LBP, exercise has the highest evidence of effectiveness in avoiding chronic disability and preventing recurrence [12]. On the other hand, there is insufficient evidence on the efficacy of thermotherapy, therapeutic ultrasound, TENS, and electrical stimulation in physical rehabilitation [12]. Guidelines for the treatment of LBP are now widely available and are used to improve patient care [13]. However, effectiveness of physiotherapy is thought to be directly related to the patients' adherence or compliance with the therapy regimen [14,15]. The World Health Organization defines adherence as "the extent to which a person's behavior such as taking medication, following a diet, executing lifestyle changes like exercising, corresponds with agreed recommendations from a health care provider [16]. Kroll and colleagues (1999)[17] defined compliance as a process in which the patient works to maintain health in close collaboration with health care providers. The term "adherence" is considered synonymous with "compliance" in the majority of studies [18-20].

There is scarce information on factors affecting adherence or compliance with physiotherapy in patients with LBP. One aspect of adherence that pertains directly to physiotherapy is attending appointments [21]. Non-adherence to treatment suggested by health care providers may have serious consequences such as exacerbation of symptoms, development or progression of disability, and failure to attain expected positive treatment outcome [22]. Attendance rate at physiotherapy is recommended as a measure of clinic-based adherence [23-25].

While the multi-dimensional problem of LBP has been studied extensively in industrialized countries [5], an understanding of how culture affects LBP is still needed in many developing countries. Furthermore, the literature suggests a specific gender-based consideration when making recommendations for improving adherence [26]. In Saudi Arabia, there is a growing need to understand how social restrains imposed upon Saudi women affect women's health and their response to current treatment methods. Since adherence is reported to be directly influenced by social and cultural factors [17], the cultural uniqueness of Saudi women warrants special attention.

The degree to which patients, especially women, in developing countries adhere to clinic- or home-based treatment programs remains unknown. This study was conducted to investigate factors related to patients' adherence to physiotherapy in a group of Saudi female patients with LBP. In particular, the study examined attendance to physiotherapy as one indicator of adherence. Factors examined included demographic factors, patients' medical condition and history, nature of LBP, and type of physiotherapy treatment administered.

## Methods

### Participants

A retrospective chart review was conducted on all female patients with LBP referred to the department of physiotherapy at a local tertiary hospital, in the period between January 2008 to January 2009. Patients are usually referred to this physiotherapy department from physiatrists, general physicians, orthopedic or neurosurgeons. All patients included in the study were discharged from physiotherapy at the time of data collection. Patients referred to physiotherapy for compound musculoskeletal problems (e.g., LBP and knee O.A.) were excluded from the study. Patients were also excluded if they were pregnant, non-Saudi, or if they were admitted to the hospital during their course of physiotherapy.

A total of 98 charts were initially reviewed, but only 60 patients were included in the study after applying the exclusion criteria.

### Measurement of adherence

This study examined one indicator of adherence: attendance at the scheduled physiotherapy sessions [23]. For the purpose of the study, the terms "adherence" is operationally defined as the extent to which patients attend their scheduled physiotherapy appointments.

Participants who attended all their scheduled appointments were classified as adherent, and those who failed to attend 2 consecutive scheduled appointments and got discharged for "no show" status were classified as non-adherent.

### Procedure

Three categories of information were defined: patients' demographics, medical history and nature of LBP, and the treatment administered. A data collection form was prepared prior to the study, and an ethical approval was obtained from the Hospital Board of Ethics. Two physiotherapists specialized in musculoskeletal rehabilitation collected the following information from the patient's files: date of referral and date of 1^st ^physiotherapy appointment (to calculate the time gap between the original referral and when the patient received physiotherapy), number of physiotherapy sessions per week, total number of physiotherapy sessions attended, and nationality of the treating physiotherapist. Details on the patient medical condition, original medical diagnosis, and associated medical problems were also collected.

Because prognosis is thought to be affected by disease onset and duration, the analysis was divided into acute and chronic LBP. The data form included a detailed section on the nature of back pain: whether it was the first episode of back pain or whether the patient had experienced back pain before, the triggering cause of pain, the nature of pain, the initial pain intensity, and the patient's perception of improvement since commencing the treatment. Finally, the physiotherapy treatment reported in the chart was classified as either: passive, active, or a combination of both, and if the treatment was progressively changed.

### Data Analysis

Descriptive statistics were generated. Bivariate non-parametric correlational analysis was undertaken to compute Spearaman Rank Correlation Coefficient. The outcome was adherence; with participants classified as adherent, or non-adherent. Factors examined included all the variables collected in the data form as listed in the above section. Contingency tables, Chi-square and Fisher Exact tests were computed to test for differences in proportions between participants characterized as adherent or non-adherent. Statistical analysis was conducted using the SPSS software version 13.0 for Windows.

## Results

### Descriptive statistics

The sample was predominantly Saudi females with a mean age of 43.8 ± 11.8 years, with 93% married, and 83% housewives (Table 1). Participants who attended their scheduled appointments were classified as adherent (40%), and those who failed to attend 2 consecutive scheduled appointments and got discharged were classified as non-adherent (60%).

**Table 1:** Descriptive Statistics

Variable		Total sample (n = 60)	Adherent group (n = 24)	Non-adherent group (n = 36)	p-value
**Age **[mean ± SD years]		43.5 ± 10.7	44.4 ± 13.7	43.5 ± 10.7	0.07
					
**Marital status **[n (%)]	Married	56 (93%)	23 (96%)	33 (92%)	0.6
	Not married	4 (7%)	1 (4%)	3 (8.3%)	
					
**Occupation **[n (%)]	Housewife	50 (83%)	23 (96%)	27 (75%)	0.1
	Employed/student	10 (17%)	1 (4%)	9 (25%)	
					
**Time from referral to 1^st ^visit **[mean ± SD days]		12.2 ± 9.3	9.8 ± 7.3	15.3 ± 16.4	0.04*
					
**Number of sessions attended **[mean ± SD]		5.6 ± 3.2	7.2 ± 3.4	4.6 ± 2.5	0.001*
					
**Report of improvement **[n (%)]	Good	18 (30%)	16 (67%)	2 (6%)	0.005*
	Fair	21 (35%)	7 (29%)	14 (39%)	
	Poor	21 (35%)	1 (4%)	20 (56%)	

(Values are means ± SD for continuous variables, and frequency and percentages for categorical variables)

The mean time between medical referral and the first physiotherapy session was 12.2 ± 9.3 days (Table 1). Treatment administered was primarily mixed active and passive physiotherapy intervention in 88% of the sample. The patient's perceived benefit of the treatment was reported to be good (30%), fair (35%), or poor (35%).

Back pain in the majority of participants (Table 2) was characterized as mechanical (71%), insidious (75%), and recurrent (88%). The majority of participants reported having pain more than 1 year (73%), but had no previous physiotherapy (70%). The remaining 12% had back pain for 6-12 months, 7% had back pain between 3-6 months, 5% had back pain for 1-2 months, and only 3% had acute pain within 1 month.

**Table 2:** Medical condition and nature of LBP in the groups

			Groups		p-value
		Total sample (n = 60)	Adherent (n = 24)	Non-Adherent (n = 36)	
**Duration of LBP**	< 1 month	3%	50.0%	50.0%	0.161
	1-2 months	5%	66.7%	33.3%	
	3-6 moths	7%	25.0%	75.0%	
	6-12 moths	12%	0%	100.0%	
	> 1 year	73%	45.5%	54.5%	
					
**Diagnosis**	Mechanical LBP	71%	37.2%	62.8%	0.339
	Neurogenic LBP	29%	47.1%	52.9%	
					
**LBP episode**	1^st ^episode	12%	57.1%	42.9%	0.279
	Recurrent	88%	37.7%	62.3%	
					
**Other Medical Problems**	No	52%	35.5%	64.5%	0.318
	Yes	48%	44.8%	55.2%	

(Data are percentages and significance levels of comparisons between the groups using Chi-square and Fisher's Exact tests)

### Association between adherence and patient demographics

Age was significantly correlated with adherence (r = 0.7, p < 0.05), indicating that older patients were more adherent to physiotherapy (Table 3). Age was moderately correlated with the initial intensity of pain (r = 0.4, p = 0.029), reflecting that older patients had higher pain scores at their first physiotherapy visit.

**Table 3:** Bivariate correlation between attendance and patient demographics

Factors	Spearman's rho	p-value
**Age**	0.7	0.021*
**Marital status**	0.08	0.417
**Occupation**	0.18	0.254
**Number of attended visits**	-0.06	0.038
**Number of scheduled visits per week**	0.02	0.294
**Initial pain intensity**	0.5	0.042*
**Time from referral to 1^st ^visit**	-0. 4	0.008*
**Report of improvement**	0.7	0.018*

Both, marital status and occupation were not associated with adherence (Table 3). This is statistically expected since the sample is homogenous in those variables; i.e., the majority of participants were married and not working.

There was a significant negative correlation between adherence and number of sessions attended (r = -0.6, p < 0.05), suggesting that patients dropped out of their treatment at the earlier sessions. There was no correlation between adherence and the number of scheduled treatment sessions per week (Table 3).

### Association between adherence and nature of back pain

The association between adherence and nature of back pain was examined using chi-square and Fisher Exact tests. Table 2 presents the rate of adherence with respect to the participants' medical condition. There were no significant differences between adherent and non-adherent participants in relation to the duration, diagnosis, and onset of back pain; whether LBP was the 1^st ^episode or recurrent. Furthermore, co-morbidity or having other medical problems did not significantly correlate with adherence. However, adherence was correlated with the initial pain intensity (Table 3).

### Association between adherence and physiotherapy

There was a mild negative correlation between adherence and time from referral to 1^st ^physiotherapy session (r = -0.4, p = 0.008), suggesting that the earlier participants were seen after their referral, the higher their adherence (Table 3). Furthermore, adherence was correlated with subjective report of improvement (r = 0.7, p < 0.01). With respect to the treatment given, there was a significantly higher rate of non-adherence among those who did not have their treatment progressively changed (Table 4). On the other hand, adherence did not correlate with the experience or nationality of the physiotherapist (Table 4).

**Table 4:** Comparison between the groups with respect to physiotherapy

			Participants		p-value
		Total sample (n = 60)	Adherent (n = 24)	Non-Adherent (n = 36)	
**P.T nationality**	Saudi	35%	29.4%	70.6%	0.225
	Non-Saudi	65%	44.2%	55.8%	
					
**Previous P.T**	No	30%	45.2%	54.8%	0.164
	Yes	70%	27.8%	72.2%	
					
**Was the treatment progressively changed?**	No	12%	22.7%	77.3%	0.034*
	Yes	88%	50.0%	50.0%	
					

(Data are percentages and significance levels of comparisons between the groups using Chi-square and Fisher's Exact tests)

## Discussion

This study was conducted to examine adherence to physiotherapy among patients with LBP. One of the most commonly reported measures of adherence in the literature is the attendance score; i.e., the proportion of appointments attended compared to those scheduled [27,28]. The high level of non-adherence in our study is consistent with an earlier study which reported that only 44% of patients completed their scheduled course of physiotherapy [29]. Recent reports also showed that non-adherence to rehabilitation among patients with LBP may be as high as 50% [18,30]. On the other hand, others suggested that the rate of adherence could be as high as 85% in patients with herniated disc disease and 89% in those with mechanical LBP [25]. All studies, including the current, agree that adherence is not related to the type or classification of back pain [18,25,30]. Discrepancy in estimating adherence level among studies may be due to using different definitions and measures of adherence.

There is a good agreement in the literature on the importance of exercise therapy in the treatment of LBP [30,31,12]. But for exercise to be of therapeutic value it has to be done regularly and consistently with patients attending all their prescribed training physiotherapy sessions [32]. Our results show that all patients were given an exercise program as an essential part of their treatment. Yet, patients dropped out of their treatment at the earlier treatment sessions. This is probably due to the fact that benefits of exercise are not attained immediately [33]. According to Friedrich and colleagues (1998)[30], combining exercise with a motivation program increases the rate of attendance at scheduled physiotherapy sessions. We suggest discussing treatment goals and objectives with the patients earlier in the treatment, since including patients in the decision-making is known to improve patient satisfaction [34]. Patients need to know that adhering to their prescribed exercise regimen will lead to symptom relief and reduced disability on the long term [35,36].

Psychosocial factors are believed to play a role in the patient's response to treatment [37,38]. However, there is little evidence in the literature for predicting non-adherence to treatment [14]. Our data show a high level of non-adherence in a group of Saudi married females. Generally, the literature suggests that males are more adherent than females [18], and married patients are more likely to adhere to treatment than unmarried patients [18].

Since the likelihood of missing treatment sessions may be affected by the patient personal circumstances [39], we suggest that lack of transportation may be a factor contributing to non-adherence in our study. By law, Saudi females are prohibited from driving and require a guardian escort for their commuting. Also, lack of time is thought to be one of the main reasons for non-adherence [15]. While this is particularly evident in western societies, interestingly the majority of participants in our study were not enrolled in an occupation, and hence lack of time may not be a major factor contributing to their non-adherence. In the Saudi culture, family obligations supersede the women's need to take care of themselves. We suggest that the hassles associated with attending treatment, such as securing transportation, play a major role in adherence among our sample of Saudi females. Further research is needed to identify the social and cultural factors influencing adherence among this population.

In the present study, older patients had higher pain scores at their first physiotherapy session. In addition, our results suggest that adherence improves as the person gets older. Clearly, this could relate to the intensity of pain increasing with age, thereby older patients may continue to attend their prescribed treatment sessions to alleviate pain. Consistently, the literature suggests higher dropout or non-adherence among younger patients [40]. Physiotherapists should place greater efforts toward motivating younger patients to continue their scheduled treatment.

Lasinger and colleagues (1994) demonstrated that patients with LBP who dropped out of physiotherapy treatment programs had longer sick leaves compared to those who completed their treatment [41]. We did not explore this factor given that Workers Compensation Legislation in Saudi Arabia does not recognize back pain as a disabling condition that warrants sick leaves. On the other hand, our study shows that the longer the time lapse between referral and the first physiotherapy session, the more likelihood of non-adherence. Therefore we suggest introducing a triage screening session as early as possible, to prevent subsequent non-adherence.

There are speculations that the attending physiotherapist's competency and experience may affect patient's treatment adherence. Poor patient adherence may stem in part from the interaction between patients and physiotherapists [42,43]. However, this study concluded that there was no correlation between patient adherence and the physiotherapist nationality or experience.

It remains unclear as to what level of adherence is required to achieve beneficial effect of treatment [44,45]. Furthermore, it remains unclear whether poor adherence is the reason for ineffective treatment outcome, or whether patients' poor adherence is the results of lack of immediate therapeutic benefits of PT treatment [28]. It is quite evident however, that early withdrawal from treatment would not allow the therapeutic benefits of the treatment to be realized [25].

The major limitation of the study relates to the operational definition of adherence, primarily due to the lack of a "gold standard" measure of adherence in the literature. The current study examined only one component of adherence; attendance to scheduled physiotherapy appointments. More investigations on factors associated with patients' adherence to their prescribed treatment program are warranted. Future evaluation of adherence should be broad enough to capture the complex and multifaceted nature of rehabilitation programs.

## Conclusion

Our results suggest significant level of non-adherence to physiotherapy in Saudi females suffering from LBP. It appears that age, pain level, and perceived improvement are related to patients' adherence to scheduled physiotherapy treatment. Further studies are needed to examine adherence in its broader context. In addition, adherence rate among Saudi males should be examined. Future research should be directed toward developing strategies and measures to improve adherence to physiotherapy.

## Competing interests

The author declares that they have no competing interests.

## Authors' contributions

The sole author of this paper has conducted the literature review, designed the methodology, executed the study, carried out the data analysis, and prepared the manuscript.

## Pre-publication history

The pre-publication history for this paper can be accessed here:

http://www.biomedcentral.com/1471-2474/11/124/prepub
